# Resilience following childhood adversity: The need for a heuristic multilevel dynamic framework

**DOI:** 10.1016/j.nsa.2024.104069

**Published:** 2024-04-18

**Authors:** Jasmin M. Pasteuning, Anouk W. Gathier, Christiaan H. Vinkers, Milou S.C. Sep

**Affiliations:** a Department of Psychiatry, Amsterdam University Medical Centers Location Vrije Universiteit Amsterdam, the Netherlands; b Department of Anatomy & Neurosciences, Amsterdam University Medical Centers, Vrije Universiteit Amsterdam, the Netherlands; c GGZ InGeest Mental Health Care, Amsterdam, the Netherlands; d Amsterdam Neuroscience, Mood, Anxiety, Psychosis, Sleep & Stress Program, Amsterdam, the Netherlands; e Amsterdam Public Health, Mental Health Program, Amsterdam, the Netherlands

**Keywords:** Resilience, Childhood adversity, Multilevel dynamic framework, Reporting checklist

## Abstract

Both Childhood Adversity (CA) and resilience are heterogeneous constructs that are differently conceptualized and operationalized across studies. CA in any operationalization is associated with mental and physical health problems across the lifespan, but some individuals fare quite well after CA exposure. The large body of studies investigating CA and its consequences includes a growing number of studies on resilience following CA exposure. However, large heterogeneity in study design and conceptualization of resilience hampers integration of findings and our understanding of positive outcomes after CA. Collaborative efforts to move the field forward have been valuable yet insufficient, as a consensus on the conceptualization and study of resilience in the context of CA is still lacking. Therefore, we postulate the need for the practical implementation of a comprehensive multilevel dynamic framework of resilience after CA, which aims to guide the integration of I) the contextual diversity of CA, II) the heterogeneity of resilience operationalization in the context of CA, and III) time dynamics of resilience after CA. We build upon earlier frameworks and postulate that CA occurs in and is impacted by different contexts, such as the direct family environment and the broader community context. Second, we show that studies on resilience following CA measure outcomes across different functional levels (inflammatory, endocrine, brain structure and function, cognitive/emotional/behavioral/social functioning, and health, well-being, and quality of life), adhere to numerous definitions, and are inconsistent in referring to the integral dynamic nature of resilience. In addition, we present a multilevel dynamic framework reporting checklist to encourage practical implementation of our framework and facilitate an explicit conceptualization and assessment of resilience during or after CA exposure. Our framework and corresponding checklist could promote transparency, facilitating the synthesis of research findings, aiding in the identification of gaps for future research, and enabling multilevel modeling on the dynamic interplay between various domains of our framework.

## Highlights

• Childhood adversity (CA) is multi-contextual.
• Resilience after CA can be differentially conceptualized and operationalized.
• Resilience after CA can be studied at various levels of functioning.
• Resilience is time-dependent and dynamic.
• Our multilevel dynamic framework and corresponding checklist can be used as a heuristic to guide future studies.

## 1 Introduction

Childhood adversity (CA) is a potent risk factor for a myriad of both physical and mental health problems across the lifespan (Bellis et al., 2019; Hughes et al., 2017). As such, there is a vast literature on the association between CA and - among others - depression and anxiety disorders (Gardner et al., 2019; Kuzminskaite et al., 2021), posttraumatic stress disorder (Messman-Moore et al., 2017; Rameckers et al., 2021), psychosis (Morgan et al., 2016), substance abuse (Moustafa et al., 2021; Zhang et al., 2020), suicidality (Angelakis et al., 2019; Miller et al., 2013), cancer (Hughes et al., 2017; Hu et al., 2021a), cardiometabolic disease (Souama et al., 2023), respiratory disease (Hughes et al., 2017; Noteboom et al., 2021) and obesity (Danese et al., 2014). Most of these detrimental sequelae have been observed in children, adolescents and adults, pointing out CA as both a transdiagnostic and developmental risk factor for poor health outcomes. However, not all individuals develop maladaptive outcomes after CA exposure. There are individuals that seem to be resistant to the negative effects of CA, who are sometimes referred to as ‘resilient’.

In the last few decades, there has been a growing number of studies on resilience in the context of CA. However, both CA and resilience are heterogeneous constructs that are differently conceptualized and operationalized in the literature. For instance, some studies define resilience as the absence of psychopathology or (mental) health/behavioral problems in the face of adversity (Guibord et al., 2011; Walsh et al., 2010; Williams et al., 2012; Yoon, 2018) whereas for other studies resilience is the retainment of competence or even excelling in one or more domains of functioning (Jaffee et al., 2007; Maples et al., 2014; Topitzes et al., 2013). In a similar vein, CA is often used interchangeably with other constructs describing stressful events during childhood, such as childhood trauma (CT), child maltreatment, adverse childhood experiences (ACEs) and early life stress (ELS). A widely used instrument to assess CA is the Childhood Trauma Questionnaire (Bernstein et al., 2003) that assesses the experience of emotional, physical, or sexual abuse or emotional or physical neglect before the age of 18. However, other stressful live events in childhood, such as parental divorce, parental loss or abandonment, domestic violence, being bullied by peers, natural disasters or war-related adversities are also often classified as CA. Not all stressful events during childhood seem to be equally associated with negative health outcomes. Several studies have shown that the experience of childhood abuse or neglect has a more profound impact on mental health problems across the lifespan when compared to other types of CA (Atzl et al., 2019; Hovens et al., 2015; Lee et al., 2020; Narayan et al., 2017; Negriff, 2020). Also, the impact of CA is assumed to depend on the frequency and age of CA exposure (Hughes et al., 2017; Hovens et al., 2015; Agorastos et al., 2014; Dunn et al., 2017, 2018; Li et al., 2022), the type of CA (Narayan et al., 2017; Dye, 2020; Källström et al., 2020), the relationship to the perpetrator (Källström et al., 2020; Ullman, 2007) and environmental risk and protective factors (Austin et al., 2020; Younas et al., 2022).

The (often implicit) heterogeneity in the literature regarding resilience after (childhood) adversity has been acknowledged for many years by scholars in the field of resilience (Walsh et al., 2010; Kalisch et al., 2017; Xu et al., 2018). This heterogeneity complicates the integration of findings and hampers scientific, clinical and societal progress with respect to the central question of how resilience can be recognized and promoted during or in the aftermath of CA. A conceptual framework aids the study of complex phenomena (like resilience after CA) by offering a theoretical basis to structure previous and future research and promoting knowledge utility. As such, in the past decades there have been collaborative efforts to move the field forward by proposing several frameworks of resilience after (childhood) adversity, considering resilience as a complex dynamic process of adaptation instead of a stable trait (Kalisch et al., 2017, 2019; Ioannidis et al., 2020; Malhi et al., 2019; Masten et al., 2023; Ungar et al., 2023). In their seminal work, Masten and colleagues postulate that resilience depends on the dynamic interplay between both external systems (e.g., family and school) and internal systems (e.g., immune system) and that resilience changes over time and is differentially impacted by developmental stage (Masten et al., 2018, 2021, 2023; Masten, 2016).

This developmental multisystem approach is also supported by other scholars, such as Ionnadis and colleagues, who additionally highlight the temporal dynamics and different trajectories of resilience functioning when discussing their complexity theory approach (Ioannidis et al., 2020) and Kalish and colleagues (2019), who propose a hybrid symptom-and-resilience-factor network model, stating that resilience during or after adversity can be deconstructed into protective factors (i.e., resilience factors) at biological, psychological, and social levels that weaken the connections between mental health symptoms (Kalisch et al., 2019). Also, an ecological approach of resilience has gained popularity in the last decades, integrating the socio-ecological context and culture of an individual or system in resilience research (Masten et al., 2023). Ungar's social-ecological model of resilience is similar to the developmental multisystem approach in the sense that resilience of a system (e.g., the individual) during or after adversity is impacted by the reciprocal relationship with other systems (e.g., family, school or neighborhood) (Ungar et al., 2013, 2023).

Although these existing resilience frameworks have made a significant contribution to the conception of resilience as a complex, multisystem dynamic process, a comprehensive heuristic tool that integrates existing frameworks and guides the investigation and reporting of resilience research is currently missing. Instead of proposing another conceptual framework of resilience, we postulate a heuristic multilevel dynamic framework with corresponding reporting checklist (see supplement 1) that can be used by fellow resilience researchers to clearly specify their conceptualization and operationalization of resilience during or after CA in a structured way. The aim is to increase transparency in order to facilitate the synthetization of research findings and help identifying gaps for future research.

## 2 The multilevel dynamic framework

**Fig. 1:**
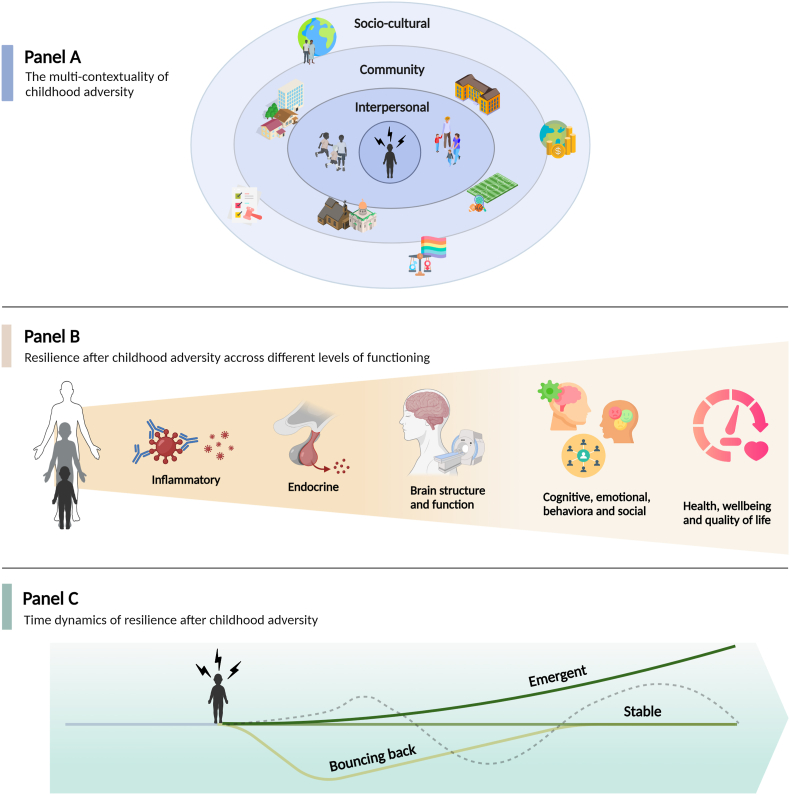
The multilevel dynamic framework of resilience after childhood adversity *Note.* Created with BioRender.com.

Our comprehensive multilevel dynamic framework is an integration of different existing resilience frameworks (i.e., developmental multilevel framework, complexity theory framework and socio-ecological framework). Because of the detrimental impact of CA on mental and physical health, we explicitly focus on resilience in the aftermath of CA instead of adversity in general. In addition, we pay attention to the different contexts in which CA can occur and be impacted to highlight the importance of the nature of CA. Our framework is based on certain axioms, which are elaborated on in the following sections and visualized in Fig. 1. Each panel of the figure represents one of the axioms, namely that CA is multi-contextual (panel A), that resilience after CA is studied and operationalized on various levels of functioning (panel B) and that resilience is not a static but a fluctuating phenomenon with time dynamics (panel C).

### 2.1 Axiom 1: CA is multi-contextual

Despite great scientific consensus about CA being an important risk factor for a range of psychiatric and somatic conditions throughout the lifespan, there is heterogeneity in findings on its prevalence and specific psychological and biological outcomes (Kuzminskaite et al., 2021). A plausible explanation for this is that CA is broadly conceptualized, as mentioned in the introduction. Moreover, the way CA is assessed can also play an important role in this heterogeneity. In fact, former studies show that there is poor agreement between prospective and retrospective measures of CA prevalence (Baldwin et al., 2019), and that retrospectively assessed CA is more strongly associated with self-reported mental and physical health problems (Berg et al., 2020; Coleman et al., 2023; Newbury et al., 2018; Reuben et al., 2016), while prospective measures of CA are more strongly associated with objective physical health problems (e.g., metabolic abnormalities, chronic inflammation and lower cortical thickness) (Reuben et al., 2016; Gehred et al., 2021; Osborn et al., 2020). In a similar vein, subjective and objective measures (e.g., official court records) of CA also differentially associate with mental health, with subjectively reported CA being more strongly associated with psychopathology than objective measures of CA (Danese et al., 2020; Francis et al., 2023). In this review, we use a broad definition of CA (including studies with subjective, objective, prospective and retrospective CA measures) as we aim to postulate a comprehensive framework of resilience after CA, incorporating study findings on events in childhood that could be experienced as traumatic (including ACEs).

Besides being a widely defined construct, CA is multi-contextual. The contexts of CA in our framework are based on Bronfenbrenner's ecological systems theory that postulates different layers of a child's environment to influence development (Bronfenbrenner, 1974; Cicchetti et al., 2015). Just like Matryoshka dolls, the ecological systems theory places the child (the smallest doll) in the center, nested within larger contexts (larger dolls) such as the interpersonal context (family and peers) that is embedded within the community context (e.g., neighborhood and school) that is in turn embedded within the larger society context (e.g., culture and norms) (Zielinski et al., 2006). In our multilevel dynamic framework, we take this socio-ecological approach to highlight the (interaction between) different environmental contexts in which CA can occur and be impacted, instead of discussing the impact of the exposome on general human development. In a recent review, Minnis and colleagues (2014) argue for a bio-exposome model to better investigate the relationship between CA and negative health outcomes (Minnis et al., 2024). In doing so, they also refer to Bronfenbrenner's ecological systems theory, describing how characteristics of different contexts can increase or buffer the negative effects of CA. Although these moderating factors (of the relation between CA and resilience) are important to increase our knowledge on resilience after CA, our aim is to provide a structure to make explicit how CA can manifest itself in different layers of the exposome, which contextual factors increase or decrease the risk of CA occurrence and how different layers of the exposome interact regarding CA occurrence.

### 2.2 Axiom 2: resilience can be differentially conceptualized and operationalized at various levels of functioning

As also depicted in the complex systems approach of Ionnnadis and colleagues (Ioannidis et al., 2020), resilience in the context of CA is studied on various (system)levels – ranging from inflammatory alterations to overall well-being – as CA can impact functioning in different systems. Such domain-specific research is paramount in advancing the field. However, important information is lost when overarching research bridging these domains is lacking. Furthermore, as described in the introduction, the concept of resilience may differ greatly across studies, with notions of absence of psychopathology in the face of adversity (Guibord et al., 2011; Walsh et al., 2010; Williams et al., 2012; Yoon, 2018), as well as retainment of competence and even excellence in one or more domains of functioning (Jaffee et al., 2007; Maples et al., 2014; Topitzes et al., 2013). In addition, resilient functioning can be studied at different time points with respect to the moment(s) of CA. Certain factors may play an important role before CA occurs, while others may be crucial in promoting resilience after CA was endured. The way in which we conceptualize and study resilience naturally impacts the conclusions we draw regarding resilient functioning after CA. Although the heterogeneity of resilience conceptualization and operationalization is not a novel claim, there is still a wide variety in definitions and assessments of resilience after CA, also in recent studies. Therefore, we believe it is important to clearly illuminate this heterogeneity, not only within psychosocial (system)levels of functioning, such as Walsh and colleagues did (Walsh et al., 2010), but also within biological (system)levels of functioning.

### 2.3 Axiom 3: resilient functioning is time-dependent and dynamic

Resilience is predominantly considered a dynamic process, rather than a static phenomenon (Kalisch et al., 2019). This means it could vary over time and cannot be perceived as a trait alone, which is often the case. This stresses the necessity to incorporate time dynamics in our multilevel dynamic framework. Heterogeneous implicit assumptions about the dynamics of functioning over time are common in resilience literature. While most studies aim to examine or promote resilience at one moment in time, they all, either implicitly or explicitly, presume its course, at the very least until that moment. Therefore, an adequate conceptualization of resilience includes an explicit description of (the presumed) time dynamics of resilience.

## 3 Axiom 1: CA is multi-contextual

In panel A of Fig. 1 we present the contexts in which CA can occur, based on Bronfenbrenner's ecological systems theory. Within each context, potential risk and protective factors could be present that increase or reduce the risk of CA exposure (Austin et al., 2020; Yule et al., 2019).

### 3.1 Individual level: characteristics of the child

With the important message that no child should ever be held responsible for being exposed to CA, there are indications that some characteristics on the child-level associate with a greater risk of CA (Austin et al., 2020). First of all, gender differences are observed in the prevalence of different CA types, with sexual abuse being more prevalent in girls/women and physical bullying by peers being more prevalent in boys/men (Stoltenborgh et al., 2015; Teicher et al., 2015). In addition, there are indications that children with disabilities, such as cognitive disabilities, physical limitations, chronic diseases and sensory impairments, are at a greater risk of experiencing CA (Fang et al., 2022; Legano et al., 2021) and that ‘sexual minorities’, such as individuals with a gay, lesbian or bisexual orientation, have an increased risk of experiencing CA compared to their heterosexual peers (Andersen et al., 2013; Friedman et al., 2011). Furthermore, genetic factors can play a role. Indeed, research has indicated a relation between risk for and resilience after CA and a variety of polymorphisms, including those within genes encoding for the serotonin transporter (Araya et al., 2009; Bukh et al., 2009; Carli et al., 2011; Cervilla et al., 2007; Coventry et al., 2010; Gillespie et al., 2005; Jacobs et al., 2006; Kim et al., 2007; Power et al., 2010; Wray et al., 2008; Zalsman et al., 2006), the glucocorticoid receptor regulating co-chaperone FKBP5 (Buchmann et al., 2014; Terock et al., 2019), the corticotrophin-releasing hormone type 1 receptor (Bradley et al., 2008), and the bèta-2 adrenergic receptor (Liberzon et al., 2014). Moreover, a meta-analysis of genome-wide association studies identified 14 independent loci associated with CA and, through Mendelian randomization, a causal role of CA on mental health but not on physical health conditions (Warrier et al., 2021).

### 3.2 Interpersonal level: family and peers

A presupposition of Bronfenbrenner's ecological model is that the most proximate social context, the family in which a child grows up, has the strongest effect on development (Bronfenbrenner et al., 2000). Therefore, it might not be surprising that CA mostly occurs within the family context with approximately 70–80% of CA being perpetrated by parents or primary caregivers, except for sexual abuse (Finkelhor et al., 2005; Gilbert et al., 2009). Findings from recent systematic and meta-analytic research on risk and protective factors for CA suggest that – among others - parental mental health issues, parental history of antisocial behavior or criminal offending, parental history of CA, parental substance abuse, marital dispute/discord, intimate partner violence and parental social isolation are important risk factors for CA occurrence (Austin et al., 2020; Younas et al., 2022; Mulder et al., 2018). Most of these risk factors apply to all forms of CA as conceptualized in these studies (physical, sexual, and emotional abuse and multiple forms of neglect) (Austin et al., 2020; Younas et al., 2022). Furthermore, low SES and economic insecurity, reflected by – among others – material hardship, housing hardship and income losses, have been associated with an increased risk of CA (Conrad-Hiebner et al., 2020; van IJzendoorn et al., 2020). An important protective factor in the family context is the presence of caregiver social support by other family members, a romantic partner or friends (Austin et al., 2020; Younas et al., 2022). Studies on risk and protective factors of CA within the family context mainly focus on the role of mothers or parenting in its general sense. Fewer studies focus on the unique role of fathers, while in the last decades, there has been an increased involvement of fathers in childrearing (Feldman, 2023). It has been suggested that fathers make an important contribution in fostering children's resilience across developmental stages (Feldman, 2023) and that daily paternal involvement with the child reduces the risk of child neglect (Lee et al., 2012). On the other hand, paternal mental or physical health problems, young paternity, paternal absence and paternal unemployment are suggested to be specific risk factors for CA (Lee et al., 2012; Metzner et al., 2017). We believe that more research into the particular role of fathers in the occurrence and impact of CA is needed, promoting preventive interventions for fathers addressing their vulnerabilities, strengths and needs that subsequently might prevent the occurrence of CA perpetration and the negative sequelae of CA. Another important point is that parents are not the only ones who can play a critical role in CA within the family context. Positive sibling relationships could buffer the negative impact of CA by providing an important source of support (Donagh et al., 2022). Yet, although often dismissed as a normal part of family life, maltreatment between siblings, such as aggression, abuse and bullying, is proposed to be one of the most common forms of violence within the family context (Meyers, 2014; Tippett et al., 2015; Wolke et al., 2012). For example, in a sample of 4237 children and adolescents in the UK, almost 46% reported being the victim of sibling aggression, such as physical aggression, verbal aggression and teasing (Tippett et al., 2015). Sibling victimization has been associated with internalizing and externalizing mental health problems in late adolescence (Toseeb et al., 2022) and adulthood (Dantchev et al., 2019a, 2019b). Also, just as exposure to interparental violence is often conceptualized as a form of CA, seeing a sibling being maltreated by a parent can also be traumatizing, resulting in fear and psychological distress (Tucker et al., 2021). In addition, the experience of receiving less support or being maltreated more relative to a sibling (‘being the black sheep’) can have an unfavorable effect on mental health, resulting in more depressive symptoms (Jensen et al., 2013; Kullberg et al., 2021).Yet, to our knowledge, most studies on CA within the family context mainly focus on perpetration by parents. Taking into account the potential maltreatment by siblings and the (relative) maltreatment of siblings is important to create a more comprehensive picture of (the impact of) CA occurrence within the family context.

Although family seems to be the most investigated context of CA, bullying or peer victimization, such as verbal and physical abuse and systematic social exclusion, has been gaining more attention as being a potential traumatic experience in childhood (Finkelhor et al., 2015; Jenkins et al., 2023). Results of meta-analytical studies suggest that around one in three children is affected by bullying and peer victimization (González-Cabrera et al., 2021; Modecki et al., 2014). Being the victim of (cyber)bullying or peer victimization has been associated with mental health problems such as depression, anxiety, suicidal ideation, suicide attempts and substance use (Moore et al., 2017). Some studies indicate that peer victimization has a unique negative effect on mental health, beyond experiences of CA within the family context (Sansen et al., 2014; Sayyah et al., 2022). In this respect, it should be mentioned that very few studies investigated CA perpetrated by siblings and adults (mostly within the family context) alongside peer victimization while there is extant evidence that CA within the family is associated with a greater risk of peer victimization and rejection (Jenkins et al., 2023; Goemans et al., 2023; Lereya et al., 2013). This is echoed by Bronfenbrenner's ecological systems theory that states that different interpersonal systems or microsystems influence each other (Bronfenbrenner, 1974).

### 3.3 Community level: neighborhood, school and other community organizations

As human relationships do not exist in a vacuum, the context in which families live and peer relationships occur is suggested to play an important role in the etiology and impact of CA. To our knowledge, at this community level, most research has been conducted on neighborhood characteristics and the school environment. Reviews of studies that looked at the association between neighborhoods and CA point to a stable and positive relationship between CA prevalence and certain neighborhood characteristics, such as neighborhood poverty, residential instability, vacant housing, unemployment rate and increased childcare burden (Coulton et al., 2007; Freisthler et al., 2015). Although less investigated than structural neighborhood characteristics, certain neighborhood social processes are also proposed to be associated with CA rates. More specifically, collective efficacy (social control and social cohesion), intergenerational closure (the extend in which a child's parent knows (the parents of) the child's friends) and the availability of neighborhood social networks are social processes found to be associated with lower rates of CA, while neighborhood physical disorder (e.g., vandalism, abandoned or burned-out buildings, graffiti and litter in the streets) and social disorder (e.g., verbal harassment on the street, the presence of gangs and drugs on the street) are social processes suggested to be associated with higher rates of CA (Freisthler et al., 2015; Molnar et al., 2016). Limitations of research on the association between neighborhood characteristics and processes and CA are that studies often differ in their definitions of certain neighborhood characteristics and usually do not look at how these characteristics exert their influence on different CA types (Freisthler et al., 2006). Moreover, the majority of studies on neighborhood factors in CA used statistical techniques at the aggregate group level that hampers our understanding of how variables on the individual, family, community and even societal level interact with each other (Maguire-Jack, 2014). When it comes to CA in the context of the school environment, being bullied or victimized by peers is the most prevalent form of CA (Menesini et al., 2017). As previously mentioned, around one in three children is affected by bullying and peer victimization (González-Cabrera et al., 2021; Modecki et al., 2014), although prevalence rates differ across studies, probably due to differences in operationalization and cultural context (Menesini et al., 2017). In line with the ecological systems theory, it is suggested that CA exposure in school is influenced by characteristics of other contextual systems, such as negative family interactions (Hong et al., 2012). When zooming in at the school context, school connectedness and other school climate factors seem to play a role in ‘school victimization’ (Hong et al., 2012; Chen et al., 2023; Gage et al., 2014). School connectedness, defined as belonging to the school environment and the feeling that both peers and teachers care about one's learning and wellbeing, is suggested to be protective against peer victimization in the sense that those who feel more connected to their school are less likely to engage in or being the victim of bullying and peer aggression (Hong et al., 2012; Chen et al., 2023; Gage et al., 2014). Other school climate factors, such as teacher support and respect for differences are also found to be negatively related to bullying/peer victimization (Gage et al., 2014).

Although CA within the family context and school environment has received more scientific and public attention, CA can also occur in (other) community institutions and organizations. Two examples that we will shortly highlight here are religious organizations and sport clubs. Involvement in religious organizations can provide social support and could help prevent CA or buffer the negative impact of CA (Yule et al., 2019; McLeigh et al., 2020). However, CA can also occur within the context of religion. Indeed, there are examples across religions and religious denominations, of which clerical child sexual abuse in the Catholic Church is the most investigated and most publicly known (Dressing et al., 2017; Rashid et al., 2019). Besides (sexual) abuse by persons with religious authority, the withholding from medical care for religious reasons and attempts to heal a child from supposed evil could also be identified as childhood neglect and abuse (Bottoms et al., 2015). Just as religious organizations, sport clubs are community institutions in which children can thrive and feel supported but also environments in which CA can occur, with those in a position of authority over young athletes, such as coaches and managers, being potential perpetrators (Fortier et al., 2020). Prevalence rates of CA in the context of sport vary across studies, probably due to the fact that there is no consensus on the definition of CA in sports (Stirling, 2009). Yet, it is proclaimed that it can manifest itself in different forms, often categorized as physical maltreatment (e.g., being hit, pushed or forced to continue training despite the presence of an injury), emotional or psychological maltreatment (e.g., being extremely criticized, humiliated or rejected on performance), sexual maltreatment (e.g., experiencing sexist jokes, being sexually exploited or abused) and neglect (e.g., being ignored regarding physical or emotional needs or being withheld from necessary medical or psychological care) (Fortier et al., 2020; Willson et al., 2022).

### 3.4 Socio-cultural level: culture, norms and policy

The socio-cultural context in which a child grows up represents our final and largest Matryoshka doll. Compared to the intrapersonal, interpersonal and community level, research on how certain socio-cultural factors influence CA occurrence is scarce (Klevens et al., 2018). In our opinion, the broader socio-cultural context is, however, of great importance as societal and cultural norms seem to shape our belief of what are acceptable behaviors towards a child and what constitutes as CA. For example, a recent exploratory survey study examined the relationship between self-reported CA and the perceived acceptability of CA (sexual, physical, and emotional abuse, exposure to domestic violence and neglect) of adults living in Cameroon, Japan, Canada and Germany (Wadji et al., 2023). Participants from Cameroon and Japan were more acceptable towards all CA types within their community when compared to Canadian and German participants. Second, results showed that, across all four countries and different cultures, those who reported greater acceptability of neglect and sexual abuse also reported higher levels of these CA types. Another study in mothers from 9 different countries (Chile, China, Greece, Iran, the Netherlands, Portugal, South Africa, Turkey, and Uruguay) examined between-country variations in maternal attitudes about what type of parenting behaviors are unacceptable and might constitute as child maltreatment (Mesman et al., 2020). Results suggested that across countries, parenting behaviors indicative of physical abuse were perceived as the most harmful and behaviors indicative of emotional neglect as least harmful. Second, there were between-country variations in the threshold for mothers to define parenting behaviors as maltreatment, with Chinese mothers reporting significantly higher thresholds and Dutch mothers reporting significantly lower thresholds compared to the other countries. In a similar vein, another survey study in 25 low and middle-income countries examined – among others – whether normative attitudes towards domestic violence and corporal punishment of children were associated with psychological and physical abuse of children (Lansford et al., 2014). Results indicated that psychological and physical violence towards children was significantly predicted by both maternal individual beliefs and countrywide norms regarding the acceptability of domestic violence and corporal punishment of children. Besides societal/cultural norms and values, other factors at the society level are also suggested to be associated with CA prevalence, such as gender inequity, lower societal education (lower overall literacy), child labor and lower country income level (Klevens et al., 2018; Viola et al., 2016).

### 3.5 Interactions between different contexts regarding (the impact of) CA occurrence

It has to be emphasized that, as human relationships do not exist in a vacuum, CA often not only occurs in one specific context. Instead, different contexts (i.e., different layers of the exposome) interact with each other (Belsky, 1980), with CA within one context often being associated with a greater risk of CA in another context (Jenkins et al., 2023; Goemans et al., 2023; Lereya et al., 2013). Also, risk and protective factors in a certain context could increase or reduce the risk of CA exposure in another context (Austin et al., 2020; Yule et al., 2019). For example, neighborhood impoverishment is suggested to contribute to higher levels of CA within families (Maguire-Jack, 2014) a particular type of CA, such as poverty, could also be a risk factor in a certain context (e.g., low socioeconomic status (SES) within the family) for other types of adversity, such as abuse and neglect. Lastly, the impact of CA can be influenced by risk and promotive factors of different (interacting) contexts. For example, a recent study by Sellars and colleagues showed that risk and promotive factors at the family level (e.g., quality of parent-child relationship), peer level (e.g., having a close friend), school level (e.g., school engagement) and neighborhood level (e.g., feeling safe on the streets) were associated with both negative mental health outcomes and resilience (i.e., “better-than-expected mental health outcomes”) after sibling victimization, respectively (Sellars et al., 2023).

To summarize, this section highlights the multi-contextual manifestation of CA. In our multilevel dynamic framework, we incorporate this by using the metaphor of the Matryoshka dolls with CA occurring in and being influenced by immediate interpersonal settings as well as broader community contexts, where societal and cultural factors seem to play a role in shaping how CA is defined and perceived.

## 4 Axiom 2: resilience can be differentially conceptualized and operationalized at various levels of functioning

Resilience after CA spans across multiple levels of functioning, as visualized in panel B of Fig. 1: inflammatory, endocrine, brain structure and function, cognitive/emotional/behavioral/social, and health, well-being, and quality of life. In this section, we discuss how studies have conceptualized and operationalized resilience across these different levels. Before doing so, we highlight the importance of developmental timing in studying and cultivating resilience. As supported by literature, the effects of CA, both in terms of risk and resilience, vary by developmental timing (Masten et al., 2018; Gee, 2021). Specifically, critical or sensitive periods marked by increased windows of neuroplasticity have been shown to be particularly important for the likelihood of the development of health problems or fostering of resilience after CA (Boyce et al., 2021). At the end of such a period, plasticity is actively dampened through molecular mechanisms in order to stabilize experience-driven learning and protect the individual from future adversity (Takesian et al., 2013). While in the context of no CA this reduces vulnerability, as healthy effects are preserved, experiencing CA during a sensitive period may “lock in” deleterious effects, making individuals more vulnerable to the development of health problems (Nelson et al., 2020). Variability as a function of developmental timing has been shown on several levels, including endocrine (Raymond et al., 2018) and epigenetic (Dunn et al., 2019) functioning, as well as the interaction between CA subtype and developmental stage (Pollmann et al., 2023). Particularly, stressor type has been shown to have distinct effects on neural development, with stressors characterized by threat during windows of increased neuroplasticity predominantly affecting fear learning, and those characterized by deprivation during such periods predominantly affecting proliferation and pruning of synaptic connections (McLaughlin et al., 2014; Sheridan et al., 2014). Importantly, resilience may still be promoted after the experience of CA during a sensitive period. Such enhancement further depends on developmental stage and multicontextual factors as described in section three (Gee et al., 2015). In addition, findings by Yoon et al. (2021) indicate that some inconsistencies in the resilience literature may be addressed by studies incorporating a developmental perspective (Yoon et al., 2021a). In their systematic review, they provide a comprehensive overview (Table 1) of the different conceptualizations and assessment methods of resilience after CA across different developmental stages (i.e., early childhood, school age, adolescence and adulthood). Therefore, developmental timing is an integral part of our framework, and we highly encourage researchers to consider and report on individuals’ life stages when conceptualizing and assessing resilience, as well as putting stressor type in the context of a particular developmental stage.

**Table 1:** Conceptualizations and operationalizations of resilience after childhood adversity.

Conceptualization	Operationalization	Examples/studies (across different levels of functioning)
A (personality) trait	Self-report questionnaires, such as the CD-RISC and the (brief) Resilience Scale.	143, 144, 145
Competent functioning	Self-report questionnaires on particular symptoms or domains of functioning. Competent functioning is often operationalized as scoring higher than one standard deviation below the mean (i.e., ‘within the normal range’/normative approach).	19, 21, 151, 152, 156, 165, 166
Positive or adaptive functioning or doing better than expected	Self-report questionnaires on particular symptoms or domains of functioning. Positive functioning is then defined as scoring within or above the normal range on a particular instrument. ‘Doing better than expected’ is operationalized as regressing the scores on the level of CA exposure. The residuals of this regression model are then used as a measure of resilience (residual approach).	21, 22, 127, 128, 129, 133, 134, 141, 152 155, 160
The absence of a psychiatric disorder	Self-report questionnaires or diagnostic interviews to determine the presence or absence of a disorder.	110, 138, 169-171
The absence of any (mental) health problems	Self-report questionnaire or interview to assess diagnostic disorders and subthreshold mental problems.	135, 173
Average or high QoL or life satisfaction	Self-report (self-created) questionnaire on perceived physical health. Half a standard deviation above the mean was considered resilient. One question about life satisfaction using a five-point Likert scale. No predefined cut-off score for being categorized as resilient.	174 175
None	–	139, 136

*Note.* CD-RISC = Connor-Davidson Resilience Scale, CA = childhood adversity, QoL = Quality of life.

### 4.1 Inflammatory

Inflammatory insights into resilience after CA are scarce. Spencer-Hwang et al. (2018) performed a qualitative study on resilience factors (RFs) in resilient centenarians and seniors who experienced CA, including community violence, economic hardship leading to food deprivation, and family dysfunction, such as loss of a parent or sibling, parental addiction, and parental physical and mental abuse (Spencer-Hwang et al., 2018). Centenarians and seniors were perceived as resilient due to their old age and lack of chronic illness. They defined RFs as factors that “[…] support adapting well in the face of adversity, stress, trauma, and so forth” and included both psychosocial as well as healthy lifestyle practices, such as diet, time outdoors, and rest. Participants were asked to report on exposure to these RFs during their childhood, defined as the first 17 years of their life. In addition, relevant information, such as during which years of childhood any RF was present, was included. Interestingly, each RF they identified among this population, including family and friends’ camaraderie and kinetic lifestyle, has been found in independent studies to have anti-inflammatory properties, such as Iower levels of interleukin 6 (IL-6) (Autenrieth et al., 2009; Costanzo et al., 2005; Yang et al., 2014), tumor necrosis factor alpha (TNF-α) (Hopps et al., 2011; Kiecolt-Glaser et al., 2005; Okun et al., 2011), and c-reactive protein (CRP) (Yang et al., 2014; Guillot et al., 2010; Nettleton et al., 2006; Reuben et al., 2003). Participants also reported continued practice and/or exposure to the same RFs they were exposed to during their childhood. Based on these results, the authors suggest that the practices of these anti-inflammatory RFs since early childhood have the potential to mitigate the negative biological effects of childhood adversities and toxic stress and may aid people to live a longer life span with less chronic disease.

Other studies into the relation between inflammation, CA, and resilience are less apparent. Findings by Miller et al. (2014) showed how a psychosocial intervention, focused on improving parenting, strengthening family relationships, and building youth competencies improved inflammation in children of low socioeconomic status (SES) (Miller et al., 2014), as seen by reduced expression of IL-1β, −6, −8, and −10, TNF-α and interferon gamma (IFN-γ) which orchestrate low-grade inflammation. While this study does not explicitly discuss resilience after CA, it does provide insight into which inflammatory markers are associated with buffering potentially traumatic events, as the intervention specifically countered harsh-inconsistent parenting, including slapping, hitting, and shouting to discipline children. Therefore, these findings have theoretical implications for research on resilience to CA, while not in fact studying resilience directly. This is in line with the authors' aim, as they do not claim to study resilience after CA. To our knowledge, research into the direct relation between inflammation and resilience after CA is lacking. Some evidence exists for the role of CRP and several cytokines in the relation between CA and resilience, as illustrated above. However, studies that specifically focus on inflammatory properties of resilience after CA are limited. Instead, intervention studies that bear theoretical implication without directly investigating resilience after CA are more common (see for instance (Nowack, 2008; Schoen et al., 2013)).

### 4.2 Endocrine

While a large body of research exists on the endocrine stress response, e.g., see de Kloet and Joëls (2023) (de Kloet et al., 2023), experimental studies on resilient endocrine functioning after CA are scant. Research on resilience exists, however, rarely in the context of CA. Instead, studies aim to identify ways to prevent clinical problems due to highly stressful environments, by employing an intervention during a stressful situation and subsequently quantifying that as promoting resilience (see for instance (Christopher et al., 2018; Hu et al., 2021b; Luthar et al., 2017)). Therefore, a clear conceptualization of resilience in the field of endocrinology is lacking. One study by Nishimi et al. (2022) did report interesting findings on the relation between resilience and diurnal salivary cortisol (Nishimi et al., 2022). They define resilience as positive psychological functioning among individuals exposed to early life adversity expected to bring about negative psychological sequalae, in line with Bonanno and Diminich (2013) (Bonanno et al., 2013) and Luthar et al. (2000) (Luthar et al., 2000). They specify that adaptation to CA reflects positive psychological health, rather than mere absence of impaired functioning. However, it remains unclear when psychological health is to be considered positive, a general trend in definitions of resilience that will return in a later section (4.4). They investigated psychological resilience in a large cohort of young adults who experienced no or low-to-high adversity and found an association between high adversity and blunted cortisol, regardless of psychological health. However, for those who experienced low adversity, better psychological health was associated with lower cortisol. Therefore, CA and psychological health seem to be independently associated with altered levels of diurnal cortisol in young adulthood.

While further examination of resilient endocrine functioning through investigation of CA and psychological health is needed, these findings add to the knowledge surrounding resilience after CA. Although research on endocrine functioning after CA in terms of risk is abundant (Raymond et al., 2018; Murphy et al., 2022; Wilkinson et al., 2011), research on endocrine functioning after CA in terms of resilience is lacking. In addition, a large part of the literature considers stress generally, instead of CA (Christopher et al., 2018; Hu et al., 2021b; Luthar et al., 2017). While these studies do provide insight into resilient endocrine functioning after CA, it can be argued to be limited. Instead of exploring how such functioning may manifest, suggestions can only be provided regarding the absence of negative CA - or stress-related consequences, as the inverse of an individual's vulnerability, omitting the possibility of positive adaptations. We believe a crucial factor to resilient endocrine functioning and resilience overall is not only the initial response of an individual to a stressor, but their recovery thereafter. Therefore, we consider employing a stress paradigm and monitoring of recovery paramount in studying resilient endocrine functioning after CA. This component of time-dynamics is imperative to resilient functioning of specific levels of our framework, as well as resilience as a phenomenon. While such a paradigm has been explored previously, for instance on the level of affect (Kuranova et al., 2020), recovery in terms of resilient endocrine functioning in people who experienced CA is currently still lacking. Therefore, we encourage future work to explore dynamic resilient functioning on each level of our framework.

### 4.3 Brain structure and function

As opposed to the two previous sections (4.1 and 4.2), more research exists on the neurobiological underpinnings of resilience after CA. The vast majority concerns neuroimaging findings, which will be covered here.

A recent review by Leal and Silvers (2021) (Leal et al., 2021) outlined the role of self- and emotion regulation in the relation between CA and resilience and suggest that self-regulation through cognitive control may help buffer negative effects of CA and contribute to neurodevelopmental resilience. Specifically, adolescents that (had) experienced adversity and were resilient across academic, social, and risk-taking domains showed increased gray matter volume in multiple dorsolateral PFC regions associated with cognitive control (Burt et al., 2016). While abuse and poverty confer the risk on weak lateral prefrontal recruitment during cognitive reappraisal, a well-studied emotion regulation strategy in imaging studies, adolescents that were exposed to CA and exhibited robust lateral prefrontal recruitment and attenuated amygdala reactivity during cognitive reappraisal were at decreased risk for anxiety (Rodman et al., 2019) and depression (McLaughlin et al., 2015). In a similar vein, increased patterns of covariation in regions related to the salience and executive control networks, and decreased GMC in temporo-parietal areas were associated with resilience, quantified as increased well-being, in people with CA exposure, including abuse, neglect, bullying, poverty, health-related traumas, and family/parent-related conflict and separation. Importantly, cognitive reappraisal mediated this relationship in participants with CA only, as compared to those without (Park et al., 2022). This further supports the indication of emotional and cognitive functioning and related brain areas as possible neural signatures of resilience. Interestingly, all the above-mentioned studies were reviewed by Leal and Silvers (2021), except for Park et al. (2022) (Park et al., 2022). However, all studies differed in their definition of resilience. Leal and Silvers (2021) quantify resilience as positive physical and mental health outcomes following CA, without clarification of what positive entails. In doing so, they explicitly state how resilience is operationalized as distinct from the inverse of an individual's vulnerability (Leal et al., 2021). However, Rodman et al. (2019), after extensive evaluation, conceptualize resilience as an absence of negative outcomes despite exposure to adversity (Rodman et al., 2019). In addition, Burt et al. (2016) define resilience as high competence despite a history of high adversity, highlighting the need for both competence and adversity to be high, without specifying when it can be considered as such (Burt et al., 2016). Compas et al. (2017) do not provide an explicit definition of resilience, but only imply that resilient individuals do not develop symptoms of psychopathology (Compas et al., 2017). Similarly, Miller et al. (2015) do not clearly define resilience but refer to it when discussing fewer depressive symptoms and less substance use, rule breaking, and aggressive behavior (Miller et al., 2015). However, further along the paper they address these resilience outcomes as “skin-deep”, because despite academic success and healthy lifestyles, the adolescents showed relatively poor cardiometabolic health, as reflected in obesity, blood pressure, and stress hormones. This emphasizes the importance of studying psychological and somatic resilience in tandem. Lastly, McLaughlin et al. (2015) provide no definition of resilience, as this was not the aim of the paper (McLaughlin et al., 2015). While comparing the results of such studies may be very fruitful, it can lead to misguided conclusions when not considering the scope and assumptions bound to each study. Furthermore, reward appears to play an important role in both endocrine and neurobiological resilient functioning. Richter et al. (2019) demonstrated reduced reward-related bottom-up activation in the ventral striatum (VS), ventral tegmental area (VTW) and hippocampus (HP) in individuals with high adversity load as compared to low adversity load (Richter et al., 2019). Interestingly, individuals with high adversity load showing high self-reported resilience, quantified as the capacity to cope effectively within the context of significant adversity and to maintain or regain mental health, were associated with an increased activation in the VTA and HP. In addition, a combination of high adversity load and high resilience traits was associated with an improved functional coupling between the VTA, VS, and HP. These results indicate that increased activity in the VTA and HP, as well as functional coupling between areas of the reward system may be potential resilience mechanisms after CA. The role of hippocampal activation is further supported by an earlier study by Van Rooij et al. (2016) on the effect of CA and the catechol-O-methyltransferase (COMT; Val/Met) polymorphism on inhibition-related hippocampal activation in resilient individuals (Van Rooij et al., 2016). This study found hippocampal activation to be negatively correlated to PTSD and depression symptoms and positively to trait resilience. Furthermore, hippocampal activation mediated the relation between CA and psychiatric risk or resilience in the Val/Val carriers, but not in the Met carrier group, thus suggesting a potential mechanism by which CA and the COMT genotype interact to in- or decrease resilience. Van Rooij and colleagues' definition of resilience is rather narrow, as they merely refer to another paper (Van Rooij et al., 2016) and state that “not everyone who experiences childhood trauma develops a psychiatric disorder; in fact, some do quite well and become high-functioning resilient individuals”. While this arguably remains ambiguous, the researchers then report having used the Connor-Davidson Resilience Scale (CD-RISC), which is widely used to assess trait resilience (Windle et al., 2011). Therefore, it can be argued that the conceptualization and operationalization of resilience are not in accordance, as the former refers to positive functional adaptation and the latter to a personal trait. Richter et al. (2019) quantify resilience as a dynamic, multidimensional process encompassing positive adaptation despite experiencing stress (Richter et al., 2019). In addition, they include regaining mental health as part of resilience, which many studies do not include or specify, but could very well be classified as a course of resilience. However, they used the RS-25 to determine resilience. In order to really examine the dynamic process of resilience, longitudinal studies that incorporate multidimensional measures are required.

Taken together, a lot of neuroimaging work has been done on resilience after CA that also encompasses a diverse range of life stages, including childhood, adolescence, and adulthood. Evidence particularly points to the role of self- and emotion regulation-related brain areas as well as increased activation in and functional coupling between areas of reward. However, as pointed out in a recent systematic review of Zhang et al. (2023), the evidence on brain structure and function in resilience research is currently not compelling, as findings are largely not consistent across studies (Zhang et al., 2023). This inconsistency may be due in part to the heterogeneity in resilience conceptualization, which highlights the importance to make differences in conceptualization more explicit and be wary of translating findings if discrepancies are too large.

### 4.4 Cognitive, emotional, behavioral, and social functioning

Studies on resilience in the context of CA at the cognitive, emotional, social and behavior level differ considerably in how they operationalize resilience. This is not surprising as these levels of human functioning are in itself quite heterogeneous. For example, cognitive functioning encompasses multiple mental abilities, such as language development, reasoning, remembering and problem solving. In order to gain a better understanding of resilience after CA, it is important to shed light on the heterogeneous conceptualization of resilience in social sciences. Before we do so, let us first consider the fact that many studies define resilience as a personal trait instead of an outcome or process of positive adaptation (Yoon et al., 2021a; Beutel et al., 2017; Schulz et al., 2014; Tlapek et al., 2017). These studies use self-report instruments that would directly assess resilience, with the CD-RISC as one of the most widely used scales (Windle et al., 2011). The CD-RISC, in which resilience is considered as a successful stress-coping ability, contains 25 items, each rated on a 5-point scale with a higher score reflecting greater resilience (Connor et al., 2003). Examples of items are “I tend to bounce back after illness, injury or other hardship” and “I think of myself as a strong person when dealing with life's challenges and difficulties”. Other often used instruments that propose to directly capture resilience as a personal trait are the Resilience Scale (Wagnild et al., 1993) and the Brief Resilience Scale (Smith et al., 2008). Defining resilience as a personal trait implies that a person's level of resilience remains rather stable over time. As other researchers in the field have also pointed out (Kalisch et al., 2017, 2019; Ioannidis et al., 2020; Malhi et al., 2019; Masten et al., 2023; Ungar et al., 2023). In our opinion, this is quite questionable as we will elaborate upon in section five. As well as the occurrence and impact of CA is influenced by interpersonal, intrapersonal, community and societal factors, we believe that resilience is a fluid construct that depends on contextual circumstances and that can change over time and developmental stage. This view is more in line with research that conceptualizes resilience as adaptive and positive functioning or maintaining/retaining competence at one or more domains of functioning or stage-salient tasks (Walsh et al., 2010; Yoon et al., 2021a). These definitions, however, raise the question of what should be considered as adaptive and positive functioning and competence. A common method in studies on resilience in the face of CA is to categorize people as resilient when they score within a non-clinical or normal range on certain instruments that assess cognitive, emotional, social or behavioral functioning or a combination of these types of functioning (e.g., scoring higher than one standard deviation below the mean). In the remainder of this section, we will refer to this method as the normative approach. Another common method to operationalize resilience is the residuals approach, first introduced by Bowes and colleagues (Bowes et al., 2010) Within this approach, functioning on a certain domain is regressed on the level of CA exposure by which the residuals of the regression model are used as a measure of resilience (Ioannidis et al., 2020; Cahill et al., 2022). In this way, ‘doing better than expected’ (i.e., residuals above the line of best fit) means being resilient. The residual approach has been widely used by scholars in the field who argue that resilience is a dynamic process that can only be measured after exposure to adversity and that severity of the adverse experience should be taken into account when determining resilience (Ioannidis et al., 2020; Bowes et al., 2010; Cahill et al., 2022; Amstadter et al., 2014; Cornwell et al., 2023; Elman et al., 2022; González-García et al., 2023; Höltge et al., 2022; Van Harmelen et al., 2017, 2021). However, due to its strong overlap with symptom severity and relatively poor predictive utility, it has been questioned whether residual scores actually reflect resilience, and not symptom severity state (Habets et al., 2024). Caution should therefore be applied when using this method.

Studies that conceptualize resilience as adaptive or competent cognitive functioning seem to be mostly conducted in children and adolescents (Yoon et al., 2021a), look at different domains of cognitive functioning, such as expressive and auditory language development (Yoon et al., 2023), verbal and nonverbal intelligence (Sattler et al., 2018) and academic adjustment (Maples et al., 2014) and used the aforementioned normative approach. Studies that examined resilience after CA on the level of emotional functioning mainly looked at emotion regulation and internalizing symptoms, assessed with – for example - subscales of the Child Behavior Checklist (CBCL) (Achenbach et al., 2000) and the Symptom Checklist-90 (SCL-90) (Derogatis et al., 1981) (Yoon et al., 2023; Lind et al., 2018). Here, individuals were classified as resilient when they scored within the normal range of selected (sub)scales (Yoon et al., 2023) or when the residuals of their symptoms/scores were positive after regressing out the effect of CA (Lind et al., 2018). On the behavioral level, studies in children often define resilience after CA as scoring within the normal range on the externalizing behavior subscale of the CBCL which assesses problem behavior, such as rule-breaking behavior and aggressive behavior (Yoon, 2018; Holmes et al., 2015). Another interesting conceptualization is that of Wright et al. (2019) who insinuate that being resilient after CA means not being engaged in violent offending in early adulthood (e.g., hurting someone badly in a fight or using or threatening to use a weapon against someone) (Wright et al., 2019). Clearly, this conceptualization of resilience is quite specific compared to the broader definition of ‘showing behavior within the normal range’, which again empathizes the heterogeneity of how resilience after CA exposure is conceptualized. Social functioning is also a domain of interest in resilience research. In the context of CA, the level of (pro)social behavior (e.g., positive interactions and good relationships with others, altruistic behavior and self-control) has been investigated as a protective factor (Holmes et al., 2015, 2018; Griese et al., 2014) but also as an indicator of resilience (Yoon et al., 2023; Sattler et al., 2018; Denckla et al., 2017; Paik et al., 2020). The latter studies have used different instruments to assess social functioning, used both the normative (Sattler et al., 2018) and the residual approach (Denckla et al., 2017) to operationalize resilience or were unclear about their definition of ‘social resilience’ (e.g., ‘having good relationships’) (Paik et al., 2020).

One could argue that adaptive functioning in one domain (i.e., cognitive, emotional, behavioral, or social) is not enough to speak of resilience after CA. After all, as also incorporated in our multilevel dynamic framework, CA often has a profound impact on multiple levels of functioning and has been associated with emotional problems, interpersonal issues, poorer cognitive skills, and high-risk behavior (Dye, 2018; Strathearn et al., 2020). Moreover, the level of functioning in one domain can influence the level of functioning in another domain. For instance, children and adolescents with behavioral problems tend to show less social competence (Hukkelberg et al., 2019). Indeed, quite some studies define resilience as showing competence in multiple domains of functioning. For example, in a longitudinal study that examined behavior, social, and cognitive development in 664 maltreated children, a child's competence in all three developmental domains was assessed at age four and six, using different age-specific instruments (Dubowitz et al., 2016). Children were considered competent in a certain domain if they scored within the normal range (i.e., scoring higher than one standard deviation below the mean) on the associated age-specific instrument. Subsequently, being resilient meant being competent in all three domains over time (so both at age four and six). According to this definition, 42% of maltreated children were resilient. In a similar vein, Yoon and colleagues (2021) examined resilient functioning changes over time in 771 adolescents with a history of CA, measuring competencies in four developmental domains: externalizing domain (behavioral problems), internalizing domain (depression, anxiety and somatic problems), social domain (prosocial behavior, feelings of loneliness, satisfaction with peer relationships) and cognitive domain (school achievement, reading and math skills) (Yoon et al., 2021b). Same as in the study of Dubowitz et al. (2016), scores higher than one standard deviation below the mean on associated instruments were indicative of competence in a specific domain and resilience was operationalized as showing competence across all four domains. Over a time period of 18 months, approximately 45% of the study sample remained resilient. Other studies used a co-called resilience index to quantify the level of resilience across multiple domains. For instance, Topitzes et al. (2013) investigated the level of resilience in young adults who were maltreated in childhood and constructed a summative resilience index ranging from zero to seven, reflecting the number of positive outcomes (Topitzes et al., 2013). These seven positive outcomes were 1) high school completion, 2) college attendance, 3) not being sentenced to a state or federal correctional institution or to a county jail for at least 30 days, 4) having a quarterly income of at least $3000, 5) no substance abuse problem from age 16 onward and no substance abuse treatment from age 18 onward, 6) no depressive symptoms in the past month, and 7) average or above future expectations about e.g., graduating from college and having a happy family life. Around 22% was considered resilient, defined as having a resilience index score of at least five (i.e., reporting five or more positive outcomes). Another example of using a resilience index/score is a study that examined the contribution of cognitive coping strategies to the resilience of 100 women who were sexually abused as a child (Kaye-Tzadok et al., 2017). Self-report instruments were used to assess social functioning, depression, satisfaction with life and positive affect. An overall resilience scale was constructed, combining the standardized scores of all self-report measures, with higher scores on this resilience scale reflecting higher resilience. Results of this study showed that women with a history of child sexual abuse were classified as less resilient than non-sexually abused women. Although we believe that (resilient) functioning after CA should be assessed across multiple levels of functioning, we can conclude from this subsection that there is still considerable heterogeneity in how resilience is conceptualized and operationalized in a multilevel framework. For example, as Russotti et al. (2021) nicely point out, different studies use different (combinations of) domains and methods to assess resilience. Moreover, there is often no clarity or consensus on which domains are more or less indicative of resilience (Russotti et al., 2021).

### 4.5 Health, well-being, and quality of life

Another approach to define and measure resilience in the context of CA is to look at the broader and complex level of health and well-being. Instead of focusing on how a person still manages to function despite CA, plenty enough studies suggest that the mere absence of (certain) (mental) health problems or disorders reflects resilience in the face of CA. Yet another, less common approach of conceptualizing resilience after CA is to look at an individual's well-being or quality of life. First, let us discuss some studies in which resilience is operationalized as the absence of a psychiatric disorder after CA exposure. In a study among 482 Palestinian children being exposed to ongoing war conditions, the occurrence and determinants of resilience was examined (Peltonen et al., 2014). The authors defined resilience as the absence of psychopathology despite being exposed to severe trauma. Yet, in their study, psychopathology was restricted to PTSD and other possible psychiatric disorders were not taken into account. According to their definition, 33% of the study sample was considered resilient, with friendship quality being an important predictor of resilience. In another study with a similar research population, both war-exposed and non-war-exposed Israelian children were longitudinally followed from early childhood to early adolescence (Motsan et al., 2021). In this study, children identified as resilient were exposed to war-trauma but did not develop PTSD from early childhood to early adolescence (49% of the war-exposed group). Depending on the focus of interest, other studies conceptualized resilience as not meeting the criteria of another disorder than PTSD, such as depression. For instance, Cisler and colleagues (2012) examined the effect of CA and depression on an emotion regulation brain network, using resting-state fMRI. The sample consisted of 38 women with or without CA and with or without a current or past diagnosis of major depressive disorder (MDD) (Cisler et al., 2013). In this study, women were identified as resilient when they were exposed to CA but did not have a lifetime diagnosis of MDD (18% of the study sample). With the limitation of having a small sample size, findings of this exploratory study suggest that resilient women have a more distributed pattern of information processing in the investigated emotion regulation network.

Other studies go even further in their conceptualization of resilience, stating that those who are resilient in the aftermath of CA do not have any mental health problems after CA exposure. One example that we would like to highlight here is a large-scale longitudinal study in the United States, the Great Smoky Mountains Study, in which children were assessed for subthreshold or fully diagnostic psychiatric problems (e.g., anxiety, mood, substance use and conduct problems) and adversity (e.g., family dysfunction, maltreatment and peer victimization) multiple times during their childhood (Costello et al., 1996). Copeland et al. (2023) recently conducted a follow-up study in 1266 participants of the Great Smoky Mountains Study to assess psychiatric disorders, substance use disorders, and functional outcomes in adulthood (Copeland et al., 2023). In this study, they examined the mental health status of participants who were identified as resilient in childhood. This childhood resilience was defined as being exposed to multiple forms of CA while never experiencing (subthreshold) psychiatric problems up to the age of 16. In total, 63 adult participants (around 12% of those who had been exposed to multiple adversities in childhood) were classified as being resilient as a child. However, these individuals with ‘childhood resilience’ still had a significantly higher risk of having an emotional disorder and worse physical health and financial or educational functioning in adulthood compared to participants who had no psychiatric problems as a child and were exposed to fewer types of adversity. Although this study's definition of resilience can be disputed, the results underline the notion that resilience is not a static construct but a dynamic process that can change over time and developmental phase. To our knowledge, significantly fewer studies conceptualize resilience as feeling satisfied or having a good quality of life. Yet, some studies did take this ‘positive wellbeing approach’. For instance, Banyard et al. (2017) looked at protective factors that promote resilience in a community sample of adolescents and adults who had been exposed to high levels of CA (Banyard et al., 2017). In this study, people were considered resilient when they reported an average or high physical health-related quality of life. This was operationalized as having a score of at least 0,5 standard deviation above the mean on a self-created measure of perceived physical health, including items such as “During the past 30 days, for about how many days have you felt very healthy and full of energy?“. The authors did not report how many persons were identified as resilient according to this definition, but concluded that certain protective factors, such as skills in emotion regulation and social support, were associated with better physical health-related quality of life. Another study that was conducted in an elderly sample of former childhood laborers used two indicators to define psychological resilience: lack of depressive symptoms and life satisfaction (Maercker et al., 2016). Life satisfaction was determined by using one question (“How satisfied are you at present with your life in general?“) that participants had to answer using a five-point Likert Scale, ranging from ‘not at all’ to ‘markedly’. Based on their results, the authors concluded that study participants had a relatively high life satisfaction (mean score of 3, range 1–5) and few depressive symptoms (mean score of 2,7, range 0–14), suggesting high resilience. It is of course disputable whether life satisfaction, and in this case resilience, can be captured with one single question. To summarize this subsection, most resilience research in the context of CA seem to focus on adaptive or competent functioning (showing better or equal levels of cognitive, emotional, behavioral and/or social functioning compared to age-specific norms) or the absence of ‘negativity’, in this case mental disorders or mental health problems. Research conceptualizing resilience as positive well-being or good quality of life is underrepresented. Moreover, while reading the existing literature on resilience after CA, we find it remarkable that, besides the study of Banyard et al. (2017) and Miller and colleagues (2015), we did not find studies that incorporate physical health when conceptualizing resilience. This is quite surprising as CA has been associated with unhealthy lifestyle behaviors (Kuzminskaite et al., 2021; Brugiavini et al., 2023) and somatic disorders, such as obesity, diabetes and cardiovascular disease (Hughes et al., 2017; Noteboom et al., 2021). As also postulated by our framework, we assume resilient functioning to also occur on biological levels (e.g., inflammatory, endocrine and brain functioning), in turn influencing not only mental health but also physical health.

Concluding this section on the multi-level manifestation and operationalization of resilience after CA, we can state that, despite important developments regarding resilience conceptualization, resilience after CA is still differently defined and assessed across studies and across levels of functioning. Table 1 presents the most often used conceptualizations, together with how such conceptualizations are operationalized and study examples. However, some studies discussed in this review are not presented in the table, as their conceptualizations of resilience are incongruent with their operationalization (Spencer-Hwang et al., 2018; Richter et al., 2019; Van Rooij et al., 2016; Paik et al., 2020; Ding et al., 2017). For instance, Richter et al. (2019) conceptualized resilient individuals as high functioning, while using the CD-RISC for operationalization. Also, quite some studies consider individuals resilient when scoring within or above the normal range on a particular instrument, with the former suggesting competent functioning and the latter positive or adaptive functioning. However, some of these studies that considered resilience as positive or adaptive functioning actually operationalized resilience as competent functioning, by referring to individuals as resilient when scoring within the normal range (Maples et al., 2014; Sattler et al., 2018). The strengths and limitations of different resilience operationalizations are nicely described by Cosco and colleagues (Cosco et al., 2019). In their research with older adults, they differentiate between psychometric-driven methods (e.g, using resilience scales) and data-driven methods (e.g., using statistical interaction effects to identify factors that buffer the relationship between adversity and a negative outcome, the aforementioned residual approach and the latent class approach) to operationalize resilience. Notwithstanding the fact that some of these methods can only be used when certain conditions are met (e.g., for the residuals approach, assumptions for linear regression should be met), the authors argue that the type of operationalization used depends on the study's aim and the nature of the variables in the study. For example, if the aim is to identify factors that might reduce negative outcomes after adversity (i.e., ‘resilience factors’), then researchers might decide to use statistical interaction effects. If, on the other hand, longitudinal data is examined to determine specific trajectories of (resilient) functioning after adversity, latent class approaches might be more appropriate. To our knowledge, no systematic research has yet been done into the impact of these different resilience operationalizations on the relationship between CA and functioning at specific levels (e.g., inflammatory and endocrine). Such research is currently difficult to conduct as many researchers do not report on or are unclear about their resilience operationalization.

## 5 Axiom 3: resilient functioning is time-dependent and dynamic

As outlined previously, resilience is dynamic rather than static. While a variety of resilience trajectories can be hypothesized, it is beyond the scope of this review to describe them all. Rather, we aim to show the importance of time dynamics by incorporating this in our multilevel dynamic framework and encourage researchers to explicitly include it in their conceptualization of resilience after CA. To do so, let us consider the most recognizable types of resilience courses from the literature which are visualized as continuous lines in panel C of Fig. 1. The alternative resilience courses that can be hypothesized are represented with a dotted line.

Firstly, individuals could display stable functioning after CA, meaning no significant changes take place either biologically, psychosocially or behaviorally following the traumatic event. This is illustrated as a straight line in panel C Fig. 1. It is important to note that stable functioning does not refer to the absence of a response on the levels within panel B of our framework when encountering stressors (e.g., absence of cortisol response). Rather, it refers to the absence of significant *change* after CA in comparison with functioning before occurrence of the adverse event. As mentioned previously, we believe that a dynamic response on the levels of our framework (panel B) may be essential for resilient functioning and needs to be investigated further. These dynamics, however, are not equal to those of resilience overall, as they may follow different patterns. Secondly, a decrease in functioning could occur following CA, after which individuals ‘bounce back’ to normal functioning levels (panel C Fig. 1). Whether or not a decrease in functioning reaches clinical levels differs between studies. Third and lastly, some individuals demonstrate emergent (or adaptive) functioning after CA, as can be illustrated by a positive slope in panel C Fig. 1.

A growing body of literature has explored resilience as a dynamic process, further highlighting its complexity. Work from Bonanno and colleagues emphasizes the importance of taking into account the natural heterogeneity of trauma reactions over time (Bonanno, 2021; Bonanno et al., 2012, 2015). In doing so, they argue for the explicit reference to temporal elements, including pre-adversity functioning, the actual aversive circumstances, and post-adversity resilient outcomes. To our knowledge, resilience over time, especially the identification of trajectories, has been studied mainly in the field of social sciences. For instance, a longitudinal study on adaptive social skills growth as a measure of resilience amongst maltreated adolescents identified four trajectories (Oshri et al., 2017). These were classified as the following: 1) unresponsive-maladaptive (55% of youth), quantified by a low intercept and small, negative slope, 2) stress-resistant (17% of youth), quantified by a high intercept and small, positive slope, 3) breakdown (17% of youth), quantified by a high intercept and large, negative slope, and 4) emergent resilience (10% of youth), quantified by a low intercept and large, positive slope. Out of the four, stress-resistant and emergent resilience can be categorized as resilience functioning. In a similar vein, Holmes et al. (2018) identified two trajectories of resilience in language development and academic functioning in children: high and stable functioning over time and low but increasing functioning over time, in line with the stress-resistant and emergent resilience courses respectively (Holmes et al., 2018). The authors, however, did not elaborate on the definition of high and low functioning. They found that children who were physically abused during preschool age or neglected during infancy/toddlerhood were less likely to display resilient functioning in terms of language/academic development. Furthermore, child prosocial skills, caregiver warmth, and caregiver cognitive stimulation predicted either resilience trajectories. Regarding sexual health after CA amongst adolescents, three trajectories were found: resilient (high intercept, neutral to positive slope), improving (low intercept, positive slope), and surviving (high intercept, negative slope) (Fava et al., 2018). While it may be argued that the improving class can also be categorized as resilient, it was not classified as such here. In addition, three trajectories identified by a recent study were labeled increasing resilience, decreasing resilience, and stable, low resilience, adhering to a different conceptualization of resilience, where it was defined as “the capacity of a dynamic system to adapt successfully through multisystem processes to challenges that threaten the function, survival, or development of the system” according to Masten and colleagues (2021, p. 524) (Sattler et al., 2023). Lastly, research examining externalizing behavior in children with CA identified three trajectories of problem behavior (Yoon, 2018). Children displaying a high-decreasing course exhibited clinical levels of externalizing behavior problems throughout all time points, with the levels of externalizing behavior problems gradually decreasing over time. Children displaying a moderate-increasing course started in the borderline range of externalizing behavior problems and showed increasing externalizing behavior problems, exceeding clinical levels over the years. Children displaying a low-stable course consistently showed low and normal levels of externalizing behavior problems. Interestingly, these trajectory terms are based on symptom courses, rather than resilient functioning.

It is important to note that, given its dynamic nature, resilience may occur, after which it disappears and reappears again, or something of the like. For instance, an individual could display stable functioning following CA, after which a decrease takes place and (clinical) symptoms arise, but then continues to improve to the point where they can be reclassified as resilient. For instance, longitudinal research on prospectively assessed reports on ACEs during the first 18 years in a large cohort of children identified seven resilience trajectories (Cahill et al., 2023). Such a longitudinal approach truly allowed for studying resilience as a dynamic process, resulting in trajectories such as ‘resilient to vulnerable to resilient’ and vice versa (for an intriguing model on longitudinal dynamic resilient functioning, see Ioannidis et al. (2020) (Ioannidis et al., 2020). As Infurna and Luthar point out: the assumptions on which resilience models are built, such as allowing for pre-adversity variability, greatly impact findings on (the commonality of) resilience trajectories (Infurna et al., 2016). Taken together, these findings not only show the variety of time dynamics associated with resilience, but also the variety in terminology of similar time courses and in methods to studying dynamic resilience. In addition, longitudinal resilience research yields insight into the distribution according to which individuals follow a certain trajectory, as well as the potentially predictive factors for development along a resilience course. However, in resilience research on other levels of our framework (panel B; outside of the social sciences) generally little attention is paid to the existence of time dynamics. In line with a lack of (adequately) defining resilience, dynamics of resilience are rarely discussed, especially in biological research. As literature described here has shown, several time courses exist, which may be influenced or promoted by separate factors. These courses are often implicit to definitions of resilience or related terms. For instance, describing factors as ‘protective’ implies prevention of the development of (clinical) symptoms. Therefore, it refers to stable or perhaps even emergent functioning. Other definitions of resilience refer to individuals becoming ‘high functioning’ after CA (see for instance Van Rooij et al. (2016) (Van Rooij et al., 2016)). Because resilience is not static, any study investigating it examines its current state. That state, however, is bound to what came before and what may come after. Therefore, it should not be perceived as an isolated situation. Individuals exhibiting stable functioning after CA may differ from those who initially decreased in functioning and then ‘bounced back’, while both can be categorized as resilient. In addition, especially when comparing different age groups, attention should not only be paid to underlying resilience trajectories, but to differences in prospective and retrospective measures of CA as well, as these have shown to exist both between and within these type of measures (Baldwin et al., 2019; Bell et al., 2018). Thus, we advise explicit evaluation of time dynamics of resilience beyond the social sciences, at every level of functioning, including interpretation of ‘high’ and ‘low’ functioning. Doing so will not only provide clarity on conflicting study findings, but also allow for the examination of dynamic specific elements. While longitudinal studies truly investigating the dynamic nature of resilience are highly encouraged, we understand these are not always feasible. Above all, our goal is for researchers to incorporate time dynamics in their conceptualization of resilience, which can be achieved by e.g., making explicit reference to the resilient period under investigation and/or collecting retrospective data.

## 6 Discussion

Despite valuable efforts to move the field forward by proposing several frameworks of resilience after CA, methods and findings remain heterogenous making integration consistently challenging. Therefore, we propose an integrated practical, heuristic framework related to resilient functioning in the context of CA addressing these issues in order to bring the field forward. Based on the current literature, there is ample evidence of CA being a multi-contextual problem, occurring within proximate interpersonal contexts and broader community contexts, with societal and cultural factors appearing to influence the perceived definition, acceptability and prevalence of CA. Research on resilience after CA should specify the context in which CA is investigated as each context has its own risk and protective factors that not only influence CA occurrence but might also influence its impact on (resilient) functioning. Although researchers might decide to examine CA in a particular context, such as within the family, we encourage them to consider other contexts, for example by gathering information about CA occurrence and risk/protective factors across diverse settings. With CA being a multi-contextual phenomenon, it is not surprising that resilience after CA bears a similar complexity, which can be explored in a variety of ways on various levels. On a more biological level, research has mainly been done on topics *related* to resilience after CA, bearing interesting implications for the field, but not directly on resilience after CA. Research often either does not include stressors, which may arguably be imperative to studying resilience, or includes stressors other than CA. Other research focuses on the prevention or improvement of symptoms, quantified as promoting resilience, rather than characterizing resilience in individuals with CA. While a lot of research exists on risk after CA, this provides only limited insight for resilience, as courses of development other than the absence of psychopathology are not considered. Within the biological field, neurobiological research seems most advanced in terms of genuine resilience research, indicating brain areas related to self- and emotion regulation as well as reward to be implicated in resilient functioning. In the social sciences, a substantial amount of multilevel resilience research exists. Although we think this is a good development, studies still differ considerably in how they operationalize resilience within this multilevel framework, looking at different (combinations of) domains and assessment methods to capture adaptive or competent functioning after CA. Also, here too, there is more focus on ill-being than on well-being, as researchers often quantify resilience as the absence of negative developments rather than positive adaptations. In addition, resilience or related topics are mainly studied within individuals, while findings on community and contextual resilience are scarce. Overall, there are shortcomings on each level of resilience functioning, marked mainly by a vast heterogeneity in the conceptualization of resilience.

Several main problems underlie the heterogeneous conceptualization of resilience. Firstly, while researchers allegedly study resilience, their work often lacks a(n) (explicit) conceptualization of what resilience entails. However, given the array of definitions that exist, making these explicit is required in order to prevent confusion and discordant findings due to heterogeneous conceptualization and operationalization of the same construct. For instance, Nishimi et al. (2021) examined the congruence of different approaches of resilience operationalization in around 1400 adults with a history of CA (Nishimi et al., 2021). Specifically, they measured resilience as a trait (CD-RISC), as the absence of distress (no or low levels of depressive and PTSD symptoms), as the absence of distress plus positive functioning (no or low levels of depressive and PTSD symptoms and high level of positive affect), and as ‘relative resilience’, reflecting individuals' deviation from distress levels predicted by CA severity. Their results showed that the four different measures were weakly to moderately correlated and yielded different resilience prevalence rates. For example, when resilience was operationalized as the absence of distress the prevalence was 23%, while this was only 12% when defined as the absence of distress plus positive affect. Without explicit and adequate descriptions of resilience, the origin of heterogeneous findings becomes harder to trace, jeopardizing correct interpretation of results.

Secondly, when researchers do define resilience, they generally do not do so adequately. As we have seen in this review, definitions typically refer to functioning that is “better than expected”, “positive”, or “high”. Without elaboration on what this entails, it remains unclear what is being examined and, therefore, how to interpret and compare results. One way to operationalize functioning that is described as positive, and particularly “better than expected” is the residuals approach to resilience (Reed et al., 2010). This method has been used across different domains of functioning and uses the residual scores of regression models to reflect individual degrees of resilient functioning, while taking CA exposure into account (Cahill et al., 2022). This approach has gained popularity because it provides a statistical method to operationalize resilience that matches its intuitive conceptual framework, namely ‘doing better than expected given a certain amount of CA exposure’. The residual approach, or similar approaches, would therefore be a good fit to clarify ambiguous definitions of resilience.

Thirdly, resilience is mostly studied as a concept of the mind, while research has shown the significant impact of CA on physical health also, including an increased risk for unhealthy lifestyle behaviors, obesity, cancer, or cardiometabolic and respiratory disease (Hughes et al., 2017; Danese et al., 2014; Kivimäki et al., 2023; Wiss et al., 2020). Currently, literature generally does not make a distinction between examining psychological and somatic resilience, which is reflected in their definition of the concept that implies resilient psychological functioning only. Thus, the importance of being resilient on a physical level is typically insufficiently acknowledged. We believe that an adequate definition of resilience should involve both a certain level of psychological as well as somatic functioning given CA exposure. While researchers can naturally choose either one to examine further, both should be recognized. Fourthly, although multilevel integrations within and across systems have been described (e.g. (Kalisch et al., 2019),) and utilized (e.g. (Boyce et al., 2021; Fritz et al., 2020; Smeeth et al., 2023),) in former resilience research, resilience after CA is still often examined at specific levels of functioning (e.g., only at the level of emotional functioning) and within specific external systems (e.g., only within the family context). Incorporating multiple contexts and levels of human functioning would do more justice to the complex, multilevel, dynamic nature of resilience after CA.

Lastly, social studies on resilience trajectories have shown that time dynamics of resilience can have important implications for an individual's development. However, time dynamics are rarely described or examined in resilience research outside of the social sciences. As was argued previously, resilience is a dynamic concept that varies over time. Therefore, its place in time at the moment of investigation should not be isolated from its previous development. As researchers have argued before, resilience in terms of recovery or bouncing back differs from sustained or stable resilience (Zautra et al., 2008, 2010). These trajectories may also be dependent on the type, duration, and context of CA. While resilience after chronic, enduring stress has been argued to differ from resilience after acute traumatic events (Schetter et al., 2011), the impact of other relevant CA factors, such as those discussed in section three, has yet to be studied. We suggest future studies be more aware of time dynamics of resilience that are inherent to their measurement of resilience at a certain moment in time. In addition, we see great potential in further longitudinal investigation of resilience trajectories that take type, duration, and context of CA into account.

Moving the field forward, a practical implementation of earlier efforts that consider resilience as a dynamic process and address the heterogeneity and complexity surrounding resilience research is needed. Our heuristic comprehensive framework, based on such earlier models, can be used as a heuristic to offer guidance in the congruence and integration of the complex facets of resilient functioning after CA. To this aim, we have developed a reporting checklist that we encourage researchers to use and add as a supplement to their work when studying resilience after CA (supplement 1), which encompasses all aspects of our multilevel dynamic framework. While not all items are expected to be incorporated into a single study, using this list could increase transparency and allow for the systematic synthetization of research findings. Doing so also enables the comparison of different conceptual frameworks, as deliberate analysis of findings according to our checklist may support one more than the other. In addition, previous work can be mapped onto our framework and checklist in order to better interpret previous findings and identify gaps for future research.

To conclude, our heuristic multilevel dynamic framework can be used and expanded upon as a way to monitor research on resilience after CA. Researchers should 1) be aware of the complexity surrounding resilience after CA, including the heterogeneity of conceptualization and operationalization and 2) be explicit and consistent about the context of CA and their definition, assessment, and outcomes of resilience, capturing its dynamic nature. Furthermore, we encourage research that spans across domains within and beyond panels. Given the complexity of both CA and resilience, multilevel modeling of the dynamic interplay between different contexts of CA, levels of resilient functioning, and components of time dynamics, could greatly advance the field.

## Funding

This work was supported by ZonMW (grant number 09150171910042).

## Declaration of competing interest

None.
