# Supplementary material for: Resilience following childhood adversity: The need for a heuristic multilevel dynamic framework
Source: Neurosci Appl. 2024 Apr 18;3:104069. doi: 10.1016/j.nsa.2024.104069 (PMC12244005; doi:10.1016/j.nsa.2024.104069)
Supplement: Multimedia component 1 [file mmc1.docx]

**The Multilevel Dynamic Framework Reporting Checklist**

**for research on resilience after childhood adversity**

JM Pasteuning & AW Gathier, CH Vinkers & MSC Sep (2024)

**Part A**

Please indicate whether the following items are addressed in the study (yes/no) with the corresponding location in the report.

| **Topic** | **Item** | **Addressed (Yes/No)** | **Location where item is reported (page number)**  *NA = not applicable  NR = not reported in the manuscript* |
| --- | --- | --- | --- |
| *Terminology* | Conceptualization of resilience e.g., “maintenance of mental health during and after CA” |  |  |
|  | Conceptualization of childhood adversity (CA) i.e., adversity/trauma types |  |  |
| *Methodology* | Study design  e.g., cross-sectional, longitudinal |  |  |
|  | Type of CA report  i.e., subjective (questionnaire or interview) or objective (e.g., court records) |  |  |
|  | Information on when CA is assessed with respect to resilience assessment, including prospective vs. retrospective assessment of CA |  |  |
|  | Information on developmental stage of CA occurrence  i.e., specific mention of age (range) |  |  |
|  | Information on pre-CA functioning |  |  |
|  | Time period during which resilience was measured and how often |  |  |

**Part B**

In the matrix below, indicate by checking (X) the appropriate boxes in the third column and specify the operationalization in the fourth column:

- In which context(s) is CA investigated in the study? At which level(s) of functioning is resilience after CA investigated in the study? Which time dynamics of resilience functioning after CA does the study assume or report on?

|  | **Context** |  | **Operationalization in study** e.g., as the context in which CA was assessed or as the context in which risk or protective factors for CA occurrence or the negative impact of CA were assessed.  If the interaction between contexts was addressed/investigated, please check all and describe interrelation here. |
| --- | --- | --- | --- |
| **The context(s) of childhood adversity (CA)**  *Axiom 1 of the multilevel dynamic framework* | Individual  Characteristics of the child |  |  |
|  | Interpersonal  Family and peers |  |  |
|  | Community  Neighborhood, school and other community organizations |  |  |
|  | Socio-cultural  Culture, norms and policy |  |  |
|  | **Level** |  | **Operationalization in study** e.g., report use of specific resilience scale or use of a data-driven method such as the residuals approach |
| **Level(s) of resilient functioning**  *Axiom 2 of the multilevel dynamic framework* | Inflammatory |  |  |
|  | Endocrine |  |  |
|  | Brain structure/function |  |  |
|  | Cognitive/emotional/behavioral/social functioning |  |  |
|  | Health/well-being/quality of life |  |  |
|  | *Other, namely:* |  |  |
|  | **(assumed) resilience trajectory after CA** |  | **Operationalization in study** i.e., growth mixture modeling or latent class growth analysis NA = not applicable |
| **Time dynamics of resilient functioning after CA** *Axiom 3 of the multilevel dynamic framework* | Stable functioning i.e., absence of significant change after CA in comparison with functioning before occurrence of the adverse event |  |  |
|  | ‘Bouncing back’  i.e., a decrease in functioning following CA, after which individuals ‘bounce back’ to normal functioning levels |  |  |
|  | Emergent functioning  i.e., an increase in functioning following CA |  |  |
|  | *Other, namely:* |  |  |
